# Corrigendum: Misplacement of images in a table including the structure of the cerebral cortex

**DOI:** 10.3352/jeehp.2018.15.12

**Published:** 2018-05-02

**Authors:** Editorial office, Journal of Educational Evaluation for Health Professions

**Affiliations:** Hallym University, Korea

[This is a correction to the article “The student experience of applied equivalence-based instruction for neuroanatomy teaching” J Educ Eval Health Prof 2016;13:32. https://doi.org/10.3352/jeehp.2016.13.32.]

The authors noticed on April 25, 2018 that there was an error of image placement in their article, “The student experience of applied equivalence-based instruction for neuroanatomy teaching” [1]. Specifically, under column B in Table 1, the second and third images (for the occipital and parietal cortices, respectively) were flipped. The correct presentation is for the second image to be alongside “parietal cortex” and the third image to be alongside “occipital cortex.” The authors further confirmed that this error was present only in the article itself, and not in the actual learning resources that were evaluated within the article.

The editorial office has confirmed this error and corrected the image placement in the article. The corrected version of Table 1 is also presented below. We apologize to our readers for not catching this error during the initial screening, review, and editorial process.

## Figures and Tables

**Table 1.** Stimuli used in resources one and two

Stimulus class	Category				
	A: structure name	B: image	C: function	D: pathology	E: anatomy
1	Frontal cortex	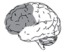	Function: selecting & planning responses	Pathology: impulsivity	Anatomy: Brodmann areas 4, 6, 8, 9, 10, 11, 44, 45
2	Occipital cortex	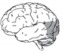	Function: vision	Pathology: sight problems	Anatomy: Brodmann areas 17, 18, 19
3	Parietal cortex	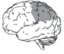	Function: attending to stimuli	Pathology: neglect	Anatomy: Brodmann areas 1, 2, 3, 5, 7, 39, 40
4	Temporal cortex	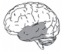	Function: identifying the nature of stimuli	Pathology: disturbed auditory perception	Anatomy: Brodmann areas 21, 22, 37, 41, 42

The four regions of the cerebral cortex constituted the classes, while the stimuli were the name (A), an image showing anatomical location (B) and information describing function (C), pathology (D), and Brodmann areas (E).
